# Attitudes of preoperative cardiac surgery patients toward COVID-19: A cause for concern

**DOI:** 10.1192/j.eurpsy.2021.1769

**Published:** 2021-08-13

**Authors:** O. Nikolaeva, E. Nikolaev, A. Zakharova, N. Maksimova, E. Litvinova, G. Dulina

**Affiliations:** 1 Cardiosurgery, Republic Cardiology Clinic, Cheboksary, Russian Federation; 2 Medical Faculty, Ulianov Chuvash State University, Cheboksary, Russian Federation; 3 Social And Clinical Psychology, Ulianov Chuvash State University, Cheboksary, Russian Federation

**Keywords:** COVID-19, cardiac surgery patients, attitudes towards COVID-19, fatalistic ideas

## Abstract

**Introduction:**

During the pandemic, many surveys studied people’s attitude to COVID-19. However, less information is available about the peculiarities of such attitude of the most vulnerable groups including chronic somatic patients who are in need of operative treatment.

**Objectives:**

To identify the specificity of preoperative cardiac surgery patients’ attitudes toward COVID-19 as compared to that of healthy people.

**Methods:**

We used the Attitudes towards COVID-19 questionnaire (Nikolaev E.) to survey 60 preoperative cardiac patients and 327 healthy university students. Difference validity was assessed with significance level of p<0.05.

**Results:**

Cardiac patients are more likely to trust the government measures to fight COVID-19 (t=3.131; p=.002), and their COVID-19-related fears for their life are higher (t=2.793; p=.005). As compared to healthy people, patients are less likely to think that pandemic broke their customary way of life (t=-2.793; p=.005) and plans for the future (t=-3.337; p=.000). Credibly more often than healthy people, cardiac surgery patients consider that it is useless to wear a mask and limit contacts, as any person will eventually catch the virus (t=2.401; p=.017). We did not reveal any more evidently expressed COVID-19-related anxiety in preoperative cardiac surgery patients.

**Conclusions:**

Attitudes of cardiac surgery patients toward COVID-19 manifest in an adequate assessment of threat to their personal health, trust in the government measures, and readiness to change their daily plans. It is latent fatalistic ideas about ultimate uselessness of restrictive measures that pose threat to people’s own health and the health of the people around them, which health professionals should remember in their preventive actions.

**Disclosure:**

No significant relationships.

